# Low-Light Image Enhancement Network Based on Recursive Network

**DOI:** 10.3389/fnbot.2022.836551

**Published:** 2022-03-10

**Authors:** Fangjin Liu, Zhen Hua, Jinjiang Li, Linwei Fan

**Affiliations:** ^1^College of Electronic and Communications Engineering, Shandong Technology and Business University, Yantai, China; ^2^Institute of Network Technology, ICT, Yantai, China; ^3^School of Computer Science and Technology, Shandong Technology and Business University, Yantai, China

**Keywords:** low light image enhancement, recursive iteration, attention mechanism, feature fusion, inception module

## Abstract

In low-light environments, image acquisition devices do not obtain sufficient light sources, resulting in low brightness and contrast of images, which poses a great obstacle for other computer vision tasks to be performed. To enable other vision tasks to be performed smoothly, it is essential to enhance the research on low-light image enhancement algorithms. In this article, a multi-scale feature fusion image enhancement network based on recursive structure is proposed. The network uses a dual attention module-Convolutional Block Attention Module. It was abbreviated as CBAM, which includes two attention mechanisms: channel attention and spatial attention. To extract and fuse multi-scale features, we extend the U-Net model using the inception model to form the Multi-scale inception U-Net Module or MIU module for short. The learning of the whole network is divided into T recursive stages, and the input of each stage is the original low-light image and the inter-mediate estimation result of the output of the previous recursion. In the *t*-th recursion, CBAM is first used to extract channel feature information and spatial feature information to make the network focus more on the low-light region of the image. Next, the MIU module fuses features from three different scales to obtain inter-mediate enhanced image results. Finally, the inter-mediate enhanced image is stitched with the original input image and fed into the *t* + 1th recursive iteration. The inter-mediate enhancement result provides higher-order feature information, and the original input image provides lower-order feature information. The entire network outputs the enhanced image after several recursive cycles. We conduct experiments on several public datasets and analyze the experimental results subjectively and objectively. The experimental results show that although the structure of the network in this article is simple, the method in this article can recover the details and increase the brightness of the image better and reduce the image degradation compared with other methods.

## 1. Introduction

**Figure 1:**
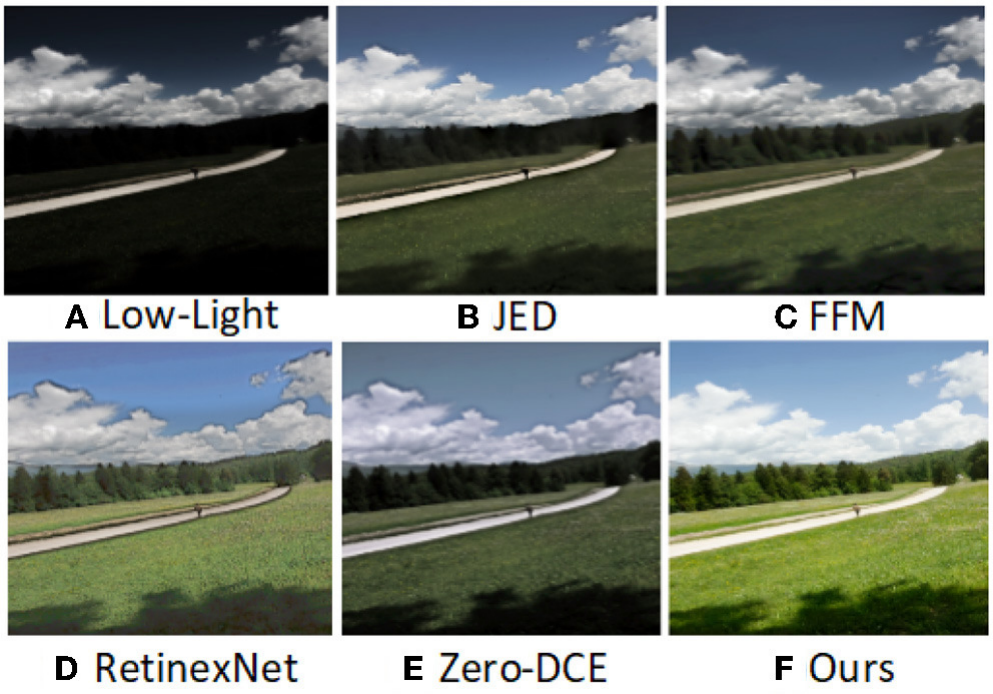
**(A)** The input low-light image. **(B-F)** are the images enhanced using JED, FFM, RetinexNet, Zero-DCE, and the method of this article, respectively.

As shown in Figure 1, images with too dark a luminance not only affects the perception of human vision but also cause great distress to other computer vision tasks. For example, for nighttime surveillance video, low illumination prevents face recognition and traffic monitoring systems from detecting the license plate number of the offending vehicle. The use of advanced camera equipment can eliminate the defect of poor quality images to some extent. However, influenced by factors such as exposure time, weather, and the intensity of scene light, images captured by advanced cameras often show blurring, high noise, excessively dark images, and color deviation. In addition, advanced camera equipment is expensive, which also brings certain limitations. The low light image enhancement method can improve the brightness and contrast of the original image and reduce the image degradation caused by various reasons. With the development of deep learning, computer vision tasks have become increasingly important. In tasks such as traffic target detection (Loh and Chan, 2018) and saliency-based image correction (Li et al., 2020), humans increasingly need high-quality images.

Therefore, for many computer vision tasks such as object recognition, object detection and image classification (Liu et al., 2020), etc. Before performing these computer vision tasks, augmenting the original images can greatly help the performance of these computer vision tasks. As shown in Figure 1, the existing low-light image enhancement algorithm has some problems. JED (Ren et al., 2018) combines two strategies of low-light image enhancement and image denoising, which decomposes the illumination and reflection maps under the condition of noise suppression, and obtains enhancement results by adjusting the illumination map. As can be seen in Figure 1, the overall brightness of the enhanced image is dark, and the outlines of distant objects are blurred. FFM (Dai et al., 2019) extracts light from low-light images, transforms dark regions into visible regions by increasing image exposure, and finally obtains more information from dark regions by fusion methods. The model effectively enhances the content of low-light regions and suppresses noise. From Figure 1, we can see that the FFM method recovers richer texture information of the object than (b), but the brightness and contrast of the image are poorer and the color is distorted. The result of the enhancement of the RetinexNet (Wei et al., 2018) method, which decomposes the low light image into reflected and illuminated components, corrects the illuminated component and then reconstructs it with the reflected component to obtain the enhanced image. As shown in (d) of Figure 1, the brightness and color of the RetinexNet method enhanced image occurred greatly, but the edges of the object were severely distorted and black artifacts appeared at the edges. Zero-DCE (Guo et al., 2020) is an unsupervised learning type of low-light image enhancement method, which enhances images with uneven colors and poor overall brightness and contrast.

**Figure 2:**
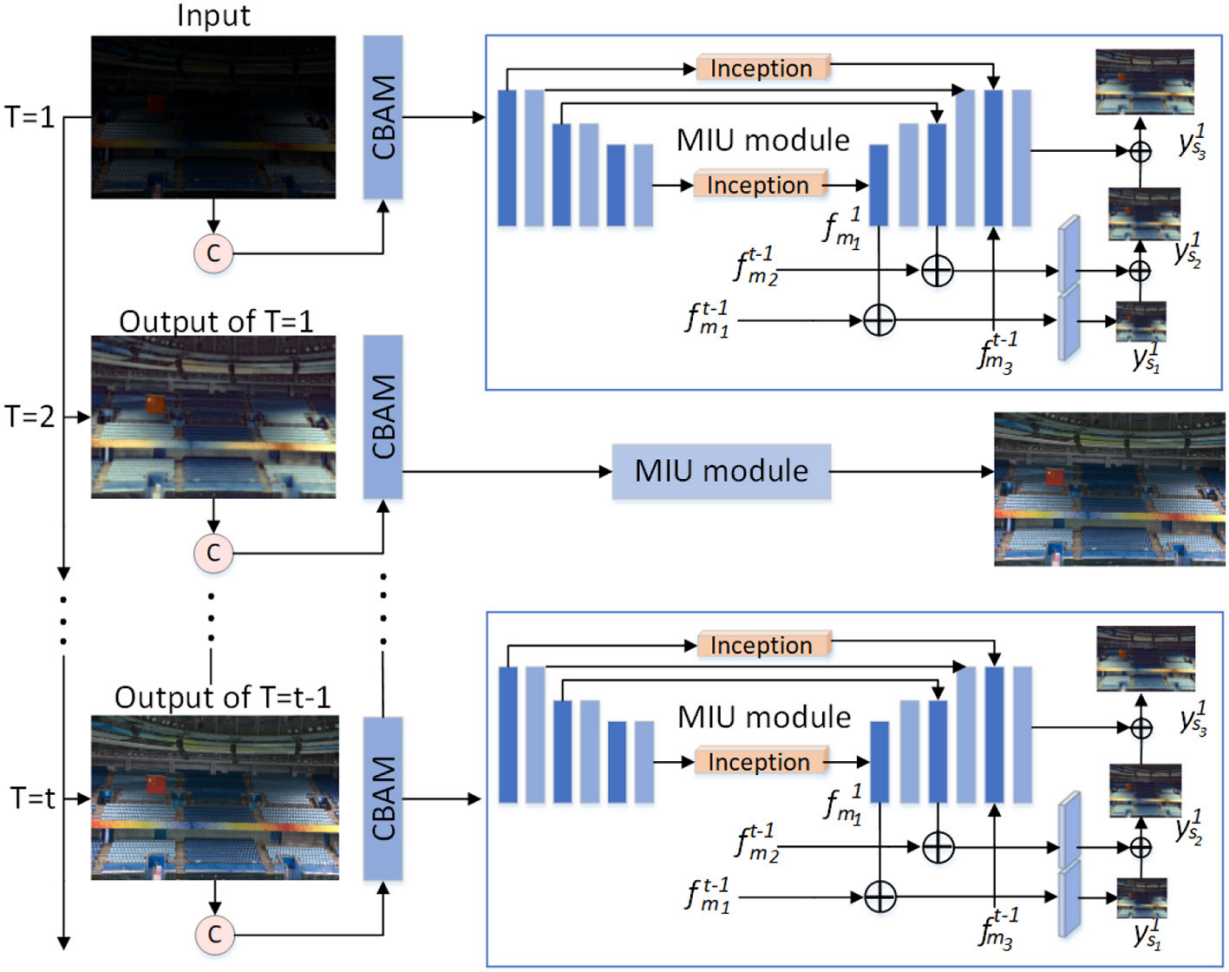
Network structure diagram. Each recursive stage inputs the original low-light image and the enhancement result of the previous recursion. Each recursive stage includes a CBAM module and an multi-scale inception U-Net (MIU) module, which extracts and fuses multi-scale features. Each recursion extracts higher-level feature information, and the final enhancement result is obtained by multiple recursions.

In order to solve the shortcomings of the above methods, we propose a low-light image enhancement method that combines the dual attention module convolutional block attentional module (CBAM) (Woo et al., 2018) and the multi-scale feature fusion module multi-scale inception U-Net (MIU). Through our method, we can obtain images with normal brightness, color fidelity, clear objects, and in line with human visual perception. Our approach is shown in Figure 2. From the paper Li et al. (2021), we learn the advantages of the recursive network. Therefore, we borrow the author's method and propose the low-light image enhancement network in this article, and our network is also based on the idea of recursion. At the *t*-th recursion, first, use the CBAM module to process the input low-light images while focusing on features in space and channel. Then, the attention feature map is entered into the MIU module. We use the inception module in the MIU module, extending the network in width to improve model accuracy and reduce parameters. Three different scales of feature maps are obtained by the MIU module. MIU module fuses multi-scale feature maps to obtain inter-mediate estimation results. The inter-mediate estimation result and the original input image are used as the recursive input for the *t* + 1′th time, and so on for iteration to complete the training learning of the network. Finally, the model is evaluated using low-light images as test data. The method in this article can repair degraded low-light images, our method effectively reduces image noise, improves color distortion, and brightness to dark. Our method is superior to several other advanced methods in the image quality evaluation index.

The contributions of our approach are mainly in the following areas:

1. A recursive low-light image enhancement network is proposed. We fuse the original input image and the enhancement result of the inter-mediate *t*-th recursion as the input for the next recursion. This allows using the previous enhancement information as a priori information. The prior information is used to correct the deficiencies in the learning process of the network at the current recursion.
2. A dual attention module, CBAM, is used to focus on information features in both spatial and channel dimensions. The module assigns different weights to channel and spatial features separately, compensating for the two shortcomings of focusing only on important features in the channel dimension or only on important features in the spatial dimension.
3. Use the inception model to extend the width and depth of the U-Net network and reduce the number of parameters but without reducing the enhancement capability of the network.
4. Contrast experiments with existing low-light image enhancement algorithms and comparisons. Experiments show that the low-light images enhanced by our method have bright and natural colors.

Next, a brief summary of related work on low-light image enhancement is presented in Section 2. The structure of the network in this article is described in Section 3. A qualitative and quantitative comparison of the network model in this article with other image enhancement methods is presented in Section 4, and the method in this article is summarized in Section 5.

## 2. Related Work

Over the years, researchers have proposed a large number of low-light image enhancement methods. We briefly outline from two angles: traditional methods and deep learning-based methods. In addition, we briefly describe the application of attention mechanisms in computer vision tasks.

### 2.1. Traditional Low Light Image Enhancement Methods

#### 2.1.1. Low-Light Image Enhancement Algorithm Based on Histogram Equalization

The histogram equalization algorithm (He et al., 2015) transforms the original histogram of an image. A non-linear transformation is used to redistribute the pixel values of the image and improve the overall contrast of the image and make the image sharper. CLAHE (Reza, 2004) algorithm sets the threshold to limit the enhancement of local contrast of the image and uses an interpolation algorithm to improve the block effect. The problem of image contrast overenhancement is solved. Chen and Ramli (2003) recursively decomposes the gray levels of the input image according to the average luminance. The resulting subhistogram is then subjected to histogram equalization. The processed image of this algorithm maintains the brightness of the image to a higher degree, but it is easy to lose the detailed information of the image. In addition, the histogram equalization algorithm has an obvious drawback, that the spatial information of the image is not considered in the image enhancement process, and the image contrast enhancement is limited.

#### 2.1.2. Low-Light Image Enhancement Algorithm Based on Wavelet Transform

Wavelet transform-based image enhancement algorithms enhance images from multiple scales. This class of algorithms considers that images acquired in low illumination environments have a greater impact on the high frequency component of the image. Jung et al. (2017) proposes an efficient contrast enhancement method based on dual-tree complex wavelet transform (DT-CWT), which can operate on a wide range of images without amplifying noise. However, most algorithms based on the wavelet transform enhance the high-frequency part of the image and ignore the low-frequency part of the image, resulting in poor enhancement.

#### 2.1.3. Low-Light Image Enhancement Algorithm Based on Retinex Theory

Retinex (Land, 1977) theory is based on color constancy. This method divides the input low-light image into light layer and reflection layer, then corrects the light layer, and finally obtains an image with uniform illumination. The multi-scale Retinex (MSR) algorithm (Jobson et al., 1997) compresses the dynamic range of the image and the brightness of the enhancement result is appropriate. But the image local area contrast is enhanced, which causes distortion of local detail color. MSRCR (Jiang et al., 2015) adds color recovery factor to MSR to adjust the color distortion problem. LIME (Guo et al., 2016) uses a prior structure to estimate the illumination map and determines reflectance for the final enhancement result. Shen et al. (2017) combining CNN with Retinex theory, a multi-scale Retinex model is proposed, which uses three functional modules of multi-scale logarithmic transformation, differential convolution, and color restoration function to enhance images. Zhang et al. (2020b) combined maximum information entropy and Retinex theory to propose a self-supervised illumination enhancement network. In addition, other algorithms based on Retinex theory in image processing can improve the image contrast. Avoiding color distortion, but the algorithms are less efficient and difficult to be applied to practical applications.

### 2.2. Low-Light Image Enhancement Algorithm Based on Deep Learning

Deep learning is a branch of machine learning. It is an algorithm that uses an artificial neural network as the architecture to perform representation learning of data. Deep learning combines low-level features to form more abstract high-level representation attribute categories or features to discover distributed feature representations of data. The motivation to study deep learning is to build a neural network that simulates the human brain for analysis and learning, which imitates the mechanism of the human brain to interpret data, such as images, sounds, and texts. So far, several deep learning frameworks, such as deep neural networks, convolutional neural networks, and deep belief networks, have been applied in the fields of computer vision, speech recognition, and natural language processing with remarkable results.

In recent years, deep learning has achieved superior results in numerous computer vision tasks, for example, image defogging (Dong et al., 2020), dynamic scene deblurring (Zhang et al., 2022). Low-light image enhancement methods based on deep learning can be divided into convolutional neural network-based methods and adversarial generative network-based methods.

#### 2.2.1. Low-Light Image Enhancement Method Based on Convolutional Neural Network

The LIME (Guo et al., 2016) algorithm achieves light map optimization by using an augmented Lagrangian number multiplication and weighting strategy. The depth autoencoder-based method LLNet (Lore et al., 2015) identifies signal features from low light images and adaptively enhances the image. The method avoids the problem of oversaturation of brighter parts of the image. RetinexNet (Wei et al., 2018) of image decomposition and successive enhancement operations. In addition, the method uses the denoising tool BM3D (Dabov et al., 2007) to denoise the decomposed reflectance map. This method gives a good decomposition scheme for image enhancement methods. KinD (Zhang et al., 2019) takes inspiration from Retinex theory. The image is decomposed into two parts, the illumination map and the reflection map, with the illumination rate used for light adjustment and the reflection rate used for degradation removal.

#### 2.2.2. Low-Light Image Enhancement Methods Based on Adversarial Generative Networks

The adversarial generative network-based approach does not require paired datasets but requires careful selection of training images. EnlightenGAN (Jiang et al., 2021) enhances images by unsupervised learning, which is based on a dual discriminator to balance global and local low-light images. Zero-DCE (Guo et al., 2020) is also an unsupervised learning approach for image enhancement. The method learns the parameters of the enhanced higher-order equation for each pixel, and the network is learned by adjusting the parameters of the equation. This method opens up a new learning strategy that eliminates the problem of the need for paired data sets, but the lack of detail recovery of the image because of the absence of real data.

In addition, some other methods were used for comparison experiments in this article. BIMEF (Ying et al., 2017) is a double exposure fusion algorithm. The algorithm learns the weight matrix for image fusion based on the illumination estimation. Guided by the weight matrix, the input image is fused with the synthetic image to obtain the enhancement result.

### 2.3. Attentional Mechanisms

The attention mechanism was first proposed in the field of visual images (Tsotsos et al., 1995). After the recurrent neural network framework was proposed, researchers combined the attention mechanism with recurrent neural networks. In 2014, the Google mind team combined the recurrent neural network model with the attention mechanism, to focus attention on a specific part of the image according to the actual demand. Similarly, when we usually observe a particular picture, we tend not to look at all factors in the picture, but to notice the salient ones first.

From Mnih et al. (2014), we know that the attention mechanism assigns high weight to relevant information and low weight to unimportant information to ignore. The mechanism continuously adjusts the weights as the network learns, and the important information can be selected in various situations. It can be classified into three categories from the perspective of the attention domain: channel attention mechanism, spatial attention mechanism, and dual attention.

The channel attention mechanism gives each channel a weight value that represents the relevance of the channel to the key information. The higher the weight, the higher the relevance, and the more attention needs to be paid to this channel. A typical network model of channel attention, SENet (Hu et al., 2018) won the ImageNet2017 classification competition. This shows that channel attention plays an important role.

Channel attention ignores the spatial structure of image regions, and the lack of spatial structure leads to inaccurate location information. This affects the accuracy of the semantic description of the generated image. In order to preserve the spatial structure of image regions, Roy et al. (2018) further proposed the spatial domain attention mechanism. The spatial domain attention mechanism uses a similar idea to the channel domain attention mechanism. Spatial attention focuses on the importance of information in different spatial locations of the feature map and finds the most important regions in the image for processing, but does not suppress other information.

Spatial attention treats the picture features of each channel equally, ignoring the information in the channel domain. Channel attention is a direct global average pooling of the information within a channel, ignoring the local information within each channel. Combining the advantages of both types of attention, a dual attention mechanism model is designed, which includes both the channel attention mechanism and the spatial attention mechanism.

Typical dual attention models are CBAM (Woo et al., 2018), bottleneck attention module (BAM) (Park et al., 2018). The CBAM model is a lightweight and general attention model. The model extracts attention maps in two dimensions, channel and space, in sequential order, respectively. The authors used CBAM for target detection and validated the effectiveness of the module using the MS COCO and VOC 2007 datasets. The experiments show that CBAM can be applied to other aspects such as classification and target detection, and the performance of different models is improved.

**Figure 3:**
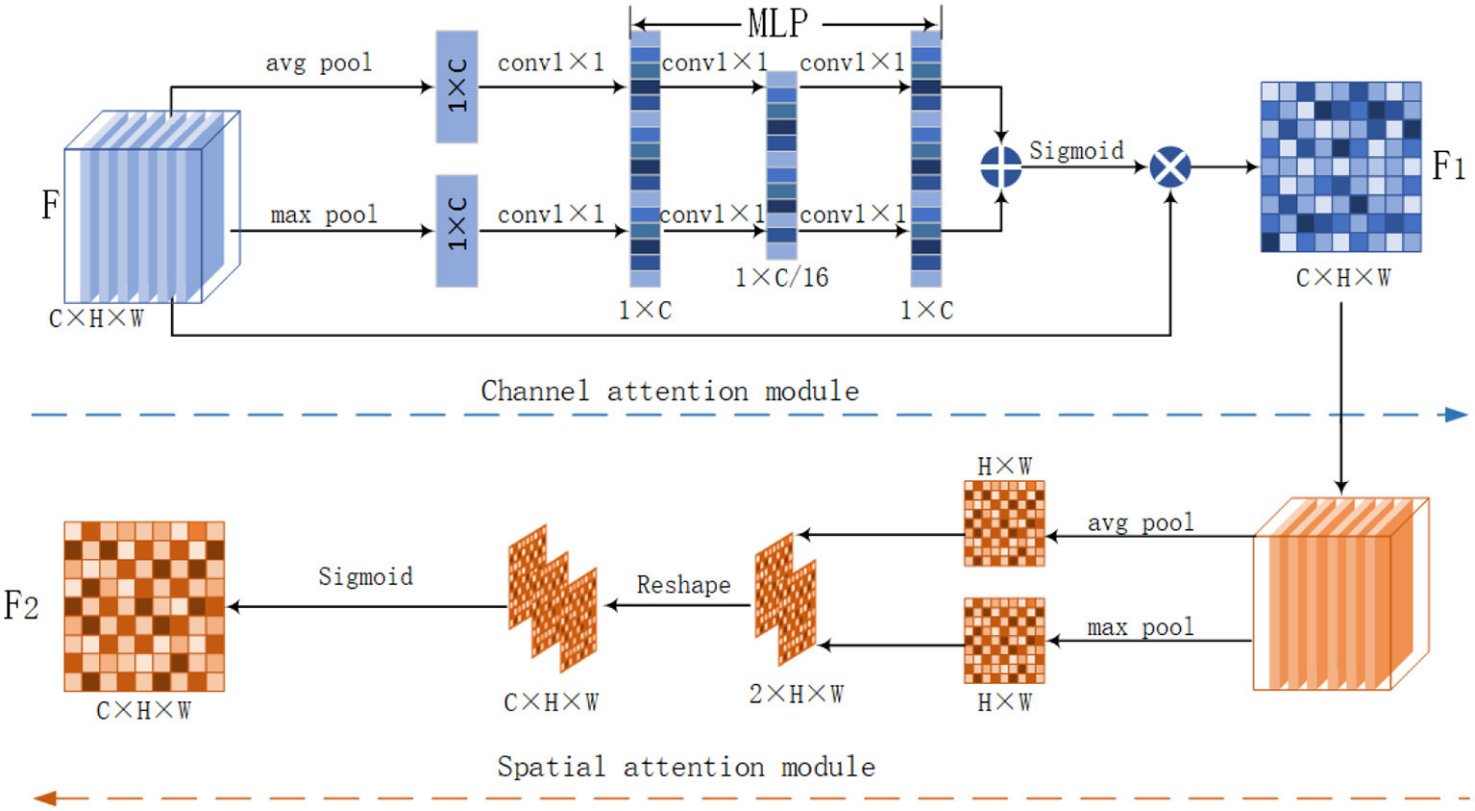
CBAM module structure diagram. The module includes a channel attention module and a spatial attention module, for the input low light image, we first use the CBAM module to extract feature attention maps.

Zhang et al. (2020a) proposed an attention-based image enhancement network that uses attention to suppress chromatic aberrations and noise, which solves the problem of the presence of severe noise in low-light images. Lv et al. (2019) found that the brightness, contrast, and noise regions of an image are closely related to underexposed regions. Thus, they used attention to distinguish between well-lit and poorly-lit regions and they used attention to distinguish between noise and true detail information. We are inspired to use the dual attention module CBAM in the approach proposed in this article, whose structure is shown in Figure 3. The CBAM module uses the channel attention module followed by the spatial attention module. The advantage of this module is that it can be embedded in any CNN network. The module fuses channel feature information and spatial feature information. The spatial attention mechanism allows the network to focus more on the more meaningful feature regions in the whole image while accelerating the convergence of the network. The channel attention mechanism allows the network to focus more on channels rich in high-frequency information during training, which can further improve the network performance.

### 2.4. Inception Module

In the field of deep learning, there are many small manipulations to improve the accuracy and generalization performance of augmented models. For example, decomposed convolution and regularization, RMSProp optimizer, Dropout, using BatchNorm, introducing residual connectivity and other related optimization ideas. Based on the above ideas, in 2014, GoogLe team proposed the Inception deep convolutional network structure in GoogLeNet (Szegedy et al., 2015). In 2015, the Google team proposed Inception V2 (Ioffe and Szegedy, 2015) and Inception V3 (Szegedy et al., 2016).

In the following year, Inception V4 was proposed (Szegedy et al., 2017). The Inception V2 model adds BN layers, reduces the internal data covariance bias, and allows the input values of each layer of neurons to be distributed as a normal distribution. This allows the input values to enter the sensitive region of the activation function and avoids gradient disappearance. In addition, the 5 × 5 convolutions in the inception module is replaced by two convolutions of size 3 × 3. The Inception V3 model divides the 7 × 7 convolution of the GoogLeNet model into two layers of 1 × 7 and 7 × 1, and similarly changes the 3 × 3 convolution to a combination of 1 × 3 and 3 × 1. Increase the non-linearity of the network and reduce the chance of overfitting. Inception V4 removes unnecessary modules.

The advantages of the inception model are simultaneous convolution at multiple scales and extraction of features to different scales. The feature information is enriched to increase the accuracy of the network. Based on Herb's theory, the inception module extracts features at multiple scales, aggregates the more relevant features together, and non-critical features are weakened to reduce redundant information. Therefore, in the low-light image enhancement network in this article, we extend the U-Net model using inception. The depth of the U-Net model is increased vertically and the width of the U-Net model is increased horizontally using the inception model. The extension is done in such a way that when making a jump connection between low-level features and high-level feature information. The low-level feature information obtained from the U-Net encoder is processed using inception V1, and then the jump connection is performed. We demonstrate through ablation experiments that the U-Net model is extended using the inception module in the image enhancement network. The image quality evaluation index PSNR is improved by 0.404.

## 3. Proposed Method

In this section, we describe the network structure in detail. First, use the CBAM module to focus on information features in both spatial and channel dimensions. Multi-scale features are then extracted using the MIU module, fusion of multi-scale features as input for the next stage of recursion. Next, we elaborate on the network model and implementation details.

In this article, we use a recursive network architecture. We design a deep network containing U-Net. We train paired image data with the aim of learning information features from coarse to fine and recovering images. The overall network architecture is shown in Figure 2. At the *t*-th recursion, the feature information is initially processed by the CBAM module for the input low-light images, which emphasizes or suppresses the information features in both spatial and channel dimensions. To increase the depth and width of the U-Net network while reducing the parameters, we introduce the classical inception module at the jump connection in the MIU module. The MIU module obtains the multi-scale feature maps and fuses the multi-scale feature maps to obtain the inter-mediate enhancement results. The inter-mediate enhancement results are used as input for the *t* + 1st recursion. In this way, the a priori feature information guides the later stage to learn the remaining image feature information and obtain a more accurate estimate. The whole network is learned in a recursive loop pattern to obtain the final enhanced image.

### 3.1. Convolutional Block Attention Module

After considering the advantages and shortcomings of both channel attention and spatial attention. We decided to use the dual attention module (CBAM) in this article. This module uses both the channel attention module and the spatial attention module to fuse the feature maps, focusing on the effective feature information in spatial and channel dimensions. During the enhancement process preserves the rich multi-dimensional information and improves the enhancement effect. The principle of action of the three attention mechanisms has been analyzed in the introduction section. The specific steps of the CBAM module are shown in Figure 3. The channel attention module of CBAM enables the neural network to automatically determine which channel is important or unimportant and assign appropriate weights to the channels.

*T*-th recursion, the input to the CBAM module is the original low-light image x and the result of the *T* − 1st recursion $y_{s_{3}}^{t-1}$. We stitch the input low-light image with the result of the previous iteration, increasing the number of feature channels, and gene-rating the feature map *F*. For the generated feature map *F*, CBAM calculates the attention *F*_2_ diagram of *F* from both channel and space dimensions. The overall structure of the CBAM module is shown in Figure 3.

The channel attention in CBAM compresses the image in spatial dimensions. The compression is performed by using a global pooling and averaging pooling compression to obtain two one-dimensional vectors. These two vectors are then processed separately by the MLP layer. MLP stands for multi-layer perceptron. MLP is often used in a 3D point cloud matching networks. MLP is similar to a fully connected layer. Each neuron in the latter layer contains all the information from the previous layer. However, unlike the fully connected layer, the multi-layer perceptron can realize the dimensional transformation and form new features. MLP realizes feature transformation and information reorganization. In this article, we use MLP to process two one-dimensional vectors. Then, the two vectors obtained are summed. Subsequently, the weight coefficients between 0 and 1 are obtained by the Sigmoid function. Finally, the weight coefficients are then multiplied with the input feature map to output the channel attention feature map.

Convolutional block attention module's spatial attention compresses the image in the channel dimension. The compression is done by using global pooling and average pooling to get two 2D image features. The 2D feature maps are stitched by channel to obtain a feature map with a channel number of 2. Then, the spatial weight coefficients are generated using the Sigmoid function. Finally, the spatial attention feature map is obtained by multiplying with *F*_1_.

First, we process the input feature map *F* in terms of width, height, and depth, that is, we perform maximum pooling and average pooling on F. We obtain the maximum pooling feature $F_{A}^{c}$ and the average pooling feature $F_{M}^{c}$. $F_{A}^{c}$ and $F_{M}^{c}$ are passed through separate MLP convolution layers. In this article, MLP includes two convolutional layers. To control the complexity of the two convolutional operations, the first convolutional layer is used as a dimensionality reduction layer. Referring to Jaderberg et al. (2015) and Park et al. (2018) et al. we set the dimensionality reduction ratio to 16 and the number of channels from 96 to 6. The second convolutional layer raises the dimensionality to the same dimension of 96 as the input features. The features generated by the MLP are summed. Then the sigmoid activation function is used for activation to obtain the feature map *C*_*c*_(*F*). Finally, the feature map *C*_*c*_(*F*) is multiplied by the original feature *F* to obtain the final channel feature map *F*_1_:


(1) $$\begin{array}{l} C_{c}\left(F\right)=\sigma \left(M\left(Ap\left(F\right)\right)+M\left(Mp\left(F\right)\right)\right)\\ =\sigma \left(w_{1}\left(w_{0}\left(F_{A}^{c}\right)\right)+w_{1}\left(w_{0}\left(F_{M}^{c}\right)\right)\right) \end{array}$$

(2) $$\begin{array}{l} F_{1}=C_{c}\left(F\right)⊗F \end{array}$$

Here, $w_{0}\epsilon R^{C/r\times C}$, $w_{1}\epsilon R^{C/r\times C}$, *w*_0_, and *w*_1_ represent the two layers of parameters in the multi-layer perceptron model. σ is the sigmoid activation function. $F_{A}^{c}$ is the average pooling feature, $F_{M}^{c}$ is the maximum pooling feature. M denotes MLP layer, AP denotes global pooling, MP denotes maximum pooling, and in order to reduce the number of parameters, the reduction rate r is set to 16 in this article, and the MLP module changes C = 96 to C/r, and finally transforms to the original channel C = 96 using the full connection. ⊗ stands for element multiplication. The spatial attention module of CBAM is shown as the spatial attention module in Figure 3. The spatial attention compresses the feature map in the channel dimension. The channel attention map F1 is used as the input feature map of the spatial attention module. Using maximum pooling and average pooling, two-dimensional feature maps $F_{A}^{s}\epsilon R^{H\times W\times 1}$ and $F_{M}^{s}\epsilon R^{H\times W\times 1}$ are obtained. $F_{A}^{s}\epsilon R^{H\times W\times 1}$ and $F_{M}^{s}\epsilon R^{H\times W\times 1}$ are stitched by channel to obtain a feature map M with channel number 2. After that, M is reduced to one channel by a 7 × 7 convolution operation. After that, M is downscaled into one channel by a 7 × 7 convolution operation and then activated by a sigmoid activation function. Finally, the input *F*_1_ is multiplied to obtain the spatial attention feature map *F*_2_:


(3) $$\begin{array}{l} S_{s}\left(F_{1}\right)=\sigma \left(f^{7\times 7}\left(\left(Ap\left(F_{1}\right),Mp\left(F_{1}\right)\right)\right)\right)\\ \;=\sigma \left(f^{7\times 7}\left(\left(F_{A}^{s}\text{,}F_{M}^{s}\right)\right)\right) \end{array}$$

(4) $$\begin{array}{l} F_{2}=S_{s}\left(F_{1}\right)⊗F_{1} \end{array}$$

**Figure 4:**
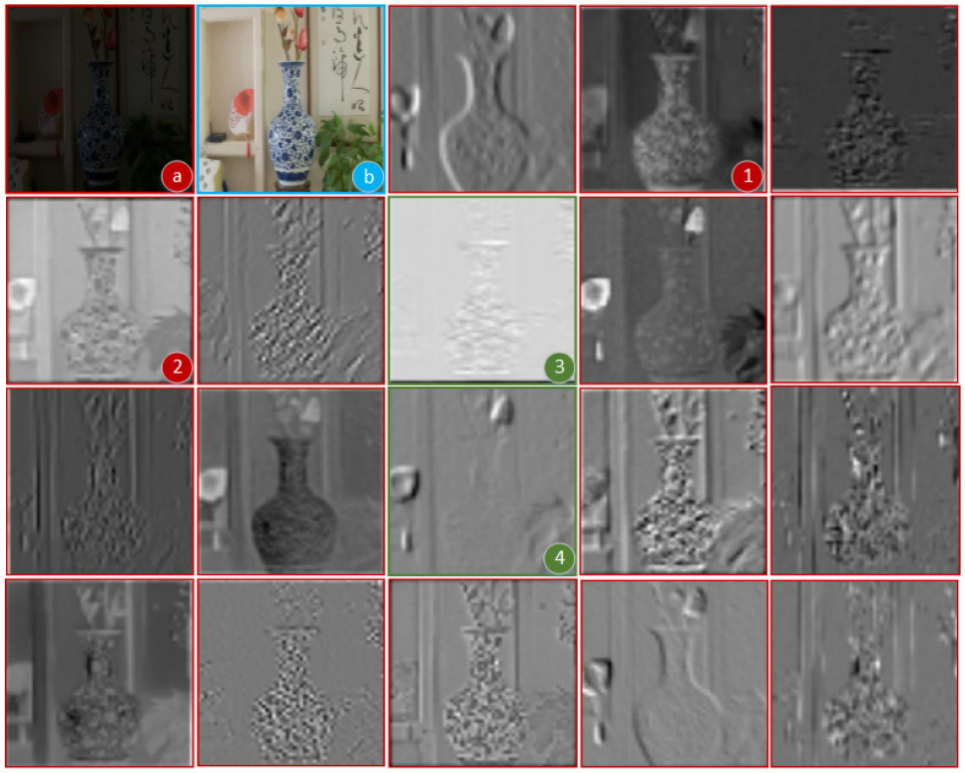
Grayscale map of each channel generated by channel attention.

In this article, we select a photograph from the low light paired dataset (LOL) (Wei et al., 2018) data training set to illustrate the role of the channel attention mechanism. This dataset is an open source dataset, collected by the authors of Wei et al. (2018). The dataset contains 500 low-light/positive-light paired images, and the image format is Portable Network Graphics format. We do not do any preprocessing before the data is used, we just use the raw low-light images directly to train the network model. First, we separate the CBAM module from the network and run it separately. A low-light image is input into the CBAM module, and the function imshow() is called to display the channel attention map, and the function imwrite() is called to save the channel attention map. The original input image is processed to have 64 channels, so 64 channel grayscale maps are output. For the convenience of representation, here we show 14 channel grayscale maps. As shown in Figure 4, a is the low light input image, b is the normal light image, and the rest are channel grayscale maps. From the introduction section, we can see that the channel attention mechanism gives a larger weight to the feature channels that are highly relevant to the visual task. For example, the shape of the vase is clearer and more obvious at red markers 1 and 2, and the texture and edges of other objects are also clearer, so it is logical that the weight of channels 1 and 2 is larger, and the weight of 3 and 4 is smaller.

### 3.2. Multi-Scale Inception U-Net

We have designed the U-Net network module in this article by referring to the ideas in the literature (Yang et al., 2020). In order to increase the performance of the whole network, but without significantly increasing the number of parameters and the computational effort. We choose to extend the width and depth of the original U-Net model. We use the inception model to increase the depth of the U-Net model vertically and the width of the U-Net model horizontally. The extension is as follows: when performing jump concatenation of encoder feature information with decoder feature information, we first use inception to process the low-level feature information obtained from the U-Net encoder and then perform the jump concatenation. The reason and role of using jump connection here is that the spatial domain information is very important in image enhancement. However, the encoder part of the U-Net network reduces the feature map resolution to a very small size by pooling layers, resulting in a large loss of spatial domain information, a situation that is not conducive to the generation of high-quality images. In the decoder part, the feature map with very small resolution can be restored to the original size by the upsampling layer. However, the upsampling process needs to fill in a lot of blank content and generate something from nothing, and this process lacks enough auxiliary information. The feature information extracted by the encoder can be referenced through a jump connection and used as auxiliary information for upsampling. These auxiliary information are semantically rich and will complement more detailed information.

In this article, the extended U-Net is referred to as the MIU module. The role of the MIU module is to obtain the multi-scale feature maps $\left[y_{s_{1}}^{T},y_{s_{2}}^{T},y_{s_{3}}^{T}\right]$ the scales of which are $\left[\frac{1}{4},\frac{1}{2},1\right]$ of the original input image, respectively. We fuse the multi-scale feature maps to obtain the inter-mediate estimation results, and the inter-mediate enhancement results are stitched with the original input image to obtain the t+1st recursive input feature map.

We illustrate the recursive implementation at t = 1 with the equation:


(5) $$\begin{array}{l} \left[f_{m_{1}}^{1},f_{m_{1}}^{1},f_{m_{1}}^{1}\right]=F_{N}^{1}\left(x\right) \end{array}$$

(6) $$\begin{array}{l} y_{s_{1}}^{1}=F_{SLU1}^{1}\left(f_{m1}^{1}\right) \end{array}$$

(7) $$\begin{array}{l} y_{s_{2}}^{1}=F_{SLU2}^{1}\left(f_{m2}^{1}\right)+up\left(y_{s_{1}}^{1}\right) \end{array}$$

(8) $$\begin{array}{l} y_{s_{3}}^{1}=F_{SLU3}^{1}\left(f_{m3}^{1}\right)+up\left(y_{s_{2}}^{1}\right) \end{array}$$

Here, $f_{m_{1}}^{1},f_{m_{2}}^{1},f_{m_{3}}^{1}$ is a multi-scale feature extracted by the MIU module; $F_{N}^{1}$ represents the process of multi-scale feature extraction; $F_{SLU1}^{1}(.)$, $F_{SLU2}^{1}(.)$, and $F_{SLU3}^{1}(.)$ denote the process of transforming multi-scale features to other corresponding scales; *up*(.) denotes the process of up-sampling the inter-mediate estimation results.

The feature map *F*_2_ output from the CBAM module is input to the MIU module, First, change the number of channels of *F*_2_ to get the first block of features *E*_1_. *E*_1_ after down-sampling the convolutional layer to obtain *E*_2_, the shape of *E*_2_ is 16 × 256 × 256. *E*_2_ continues down-sampling to get *E*_3_, that is, the third green feature block, the shape of *E*_3_ is 16 × 128 × 128. *E*_4_ is the sixth feature map in the figure, the shape of *E*_4_ is 32 × 64 × 64. Similar to other U-Net networks, up-sampling and hopping connections are performed after feature down-sampling is completed.

During the jump connection, use the Inception module to get $f_{m1}^{1}$. Then perform a jump connection on $f_{m1}^{1}$ and the result of the jump is still denoted as $f_{m1}^{1}$. The black plus sign in Figure 2 indicates a jump connection. Use Equation (6) for $f_{m1}^{1}$ to do size conversion, the result from size conversion is to produce the intermediate enhancement result $y_{s_{1}}^{1}$. The size of $y_{s_{1}}^{1}$ is the size of the original input image $\frac{1}{4}$. Perform upsampling on $f_{m1}^{1}$ and jump connect the result of upsampling with *E*_3_. From the jump connection, we obtain $f_{m2}^{1}$. Next, using Equation (7) to process $f_{m2}^{1}$ and $y_{s_{1}}^{1}$. By Equation (7), we obtain an intermediate enhanced image $y_{s_{2}}^{1}$, the size of which is $\frac{1}{2}$ of the original input size. Finally, use the Inception module again to process *E*_1_, *E*_2_. Perform upsampling on $f_{m2}^{1}$, the upsampling results of $f_{m2}^{1}$ and *E*_1_, *E*_2_ are jump connected to obtain $f_{m3}^{1}$. Using Equation (8) to process $y_{s_{2}}^{1}$ and $f_{m3}^{1}$. By Equation (8), an intermediate enhanced image $y_{s_{3}}^{1}$ is obtained, the size of which is the original input image size. From Equations (6)–(8), it can be seen that $y_{s_{2}}^{1}$ and $y_{s_{3}}^{1}$ both contain the estimated images of the previous level. This feature fusion method makes the high-order features contain the feature information of the image of the previous level. The previous-level features serve as prior information to guide the learning of higher-order features. Since we are inspired by Zhang et al. (2021), we use the above feature fusion method. In many computer vision tasks, prior feature information can guide the learning of high-order features, which improves the learning ability of the network.

After that, at the t-th iteration, the result of (t-1)-th iteration $y_{s_{3}}^{t-1}$ and the original low-light image are used as inputs:


(9) $$\begin{array}{l} \left[\Delta f_{m_{1}}^{t},\Delta f_{m_{2}}^{t},\Delta f_{m_{3}}^{t}\right]=F_{N}^{t}\left(x,y_{s_{3}}^{t-1}\right) \end{array}$$

(10) $$\begin{array}{l} f_{m_{i}}^{t}=\Delta f_{m_{i}}^{t}+f_{m_{i}}^{t-1},i=1,2,3 \end{array}$$

(11) $$\begin{array}{l} y_{s_{1}}^{t}=F_{SLU1}^{t}\left(f_{m1}^{t}\right) \end{array}$$

(12) $$\begin{array}{l} y_{s_{2}}^{t}=F_{SLU2}^{t}\left(f_{m2}^{t}\right)+up\left(y_{s_{1}}^{t}\right) \end{array}$$

(13) $$\begin{array}{l} y_{s_{3}}^{t}=F_{SLU3}^{t}\left(f_{m3}^{t}\right)+up\left(y_{s_{2}}^{t}\right) \end{array}$$

Our goal is to recover high quality images with normal brightness and clear objects in low light images. In the training process, we select some images from the LOL (Wei et al., 2018) dataset for training, and the training loss function is given as follows:


(14) $$\begin{array}{l} Loss=-g\left(y_{s_{3}}^{T},R\right)-a_{1}g\left(y_{s_{2}}^{T},↓\left(R,s_{2}\right)\right)\\ \;-a_{2}g\left(y_{s_{1}}^{T},↓\left(R,s_{1}\right)\right) \end{array}$$

Here, *R* represents a normal illuminated image with real labels, ↓ represents the down-sampling process, *s*_i_ represents the down sampled size scale, *g*(.) calculates the SSIM value of the input image, and *a*_1_, *a*_2_ is the weighting parameter.

### 3.3. Inception Module

**Figure 5:**
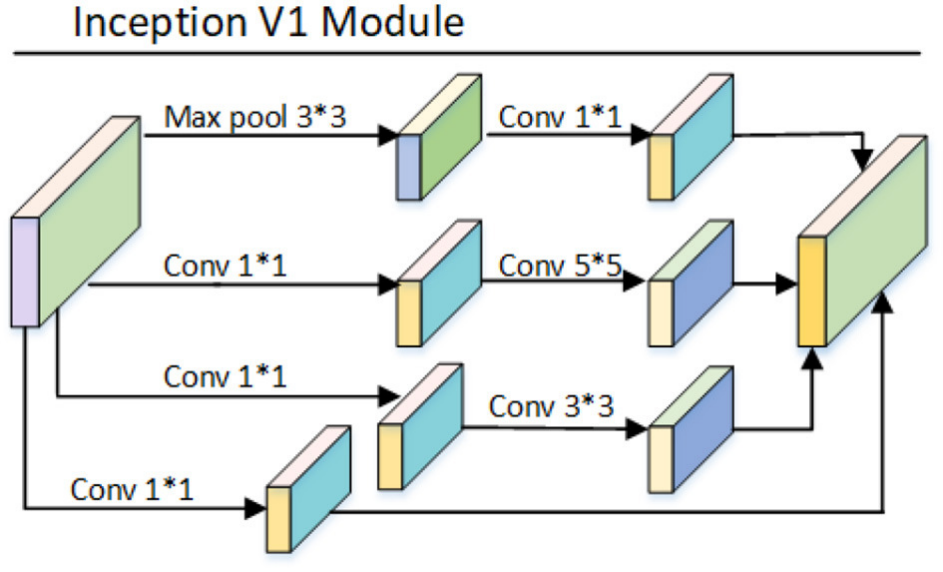
Inception V1 model structure diagram.

The main idea of the inception model is to combine and arrange the existing convolutions in an optimal way, e.g., 1 × 1, 3 × 3, 5 × 5 convolutions and find this optimal combination arrangement to approximate and cover the optimal local sparse structure of the neural network. The overall structure of the model is shown in Figure 5.

The basic process of Inception V1 model: The model uses four branches, each branch consists of 1 × 1 convolution, 3 × 13 convolution, 5 × 5 convolution, and 3 × 3 maximum pooling, all convolutions are made with ReLu activation function, combining convolution layers of different scales, increasing the width of the network and also increasing the applicability of the network to different scales, and the four branches are output and then in the channel dimension are superimposed on the channel dimension as the input to the next layer. Lin et al. (2013) proposed a 1 × 1 convolutional layer in network, which aims to reduce the number of parameters. Unlike the fully-connected layer, 1 × 1 convolution does not destroy the spatial structure of the image. The size of the input image using the fully-connected layer needs to be fixed, however, the size of the input image of the 1 × 1 convolution layer is arbitrary. Unlike the pooling layer, 1 × 1 convolution does not change the width and height of the input features, but only the number of channels. Initially, GoogleNet consisted of 9 inception modules stacked on top of each other. With the stacking of modules, the number of 3 × 3 convolutions also increased, and the computation increased dramatically. The network experienced a computational explosion after a few periods of learning. In order to solve the problem of the dramatic increase in computation. Inspired by Lin et al. (2013), a 1 × 1 convolution was introduced in the inception model, which works as follows: for the input features, the use of 1 × 1 convolution makes the number of channels decrease and removes the computational bottleneck. Making the 3 × 3 convolutions and 5 × 5 convolutions significantly less computational. The combination of 1 × 1, 3 × 3 and 1 × 1, 5 × 5 constitutes a more efficient convolution layer that can acquire more complex feature patterns.

## 4. Experimental Analysis

In this section, first, we present some implementation details of the method implementation. Then, a subjective evaluation is performed on real low-light images with other low-light image enhancement methods, and after that, we perform an objective analysis using image quality evaluation metrics. Finally, the rationality of the method in this article is demonstrated by ablation experiments.

### 4.1. Experimental Setting and Data

**Figure 6:**
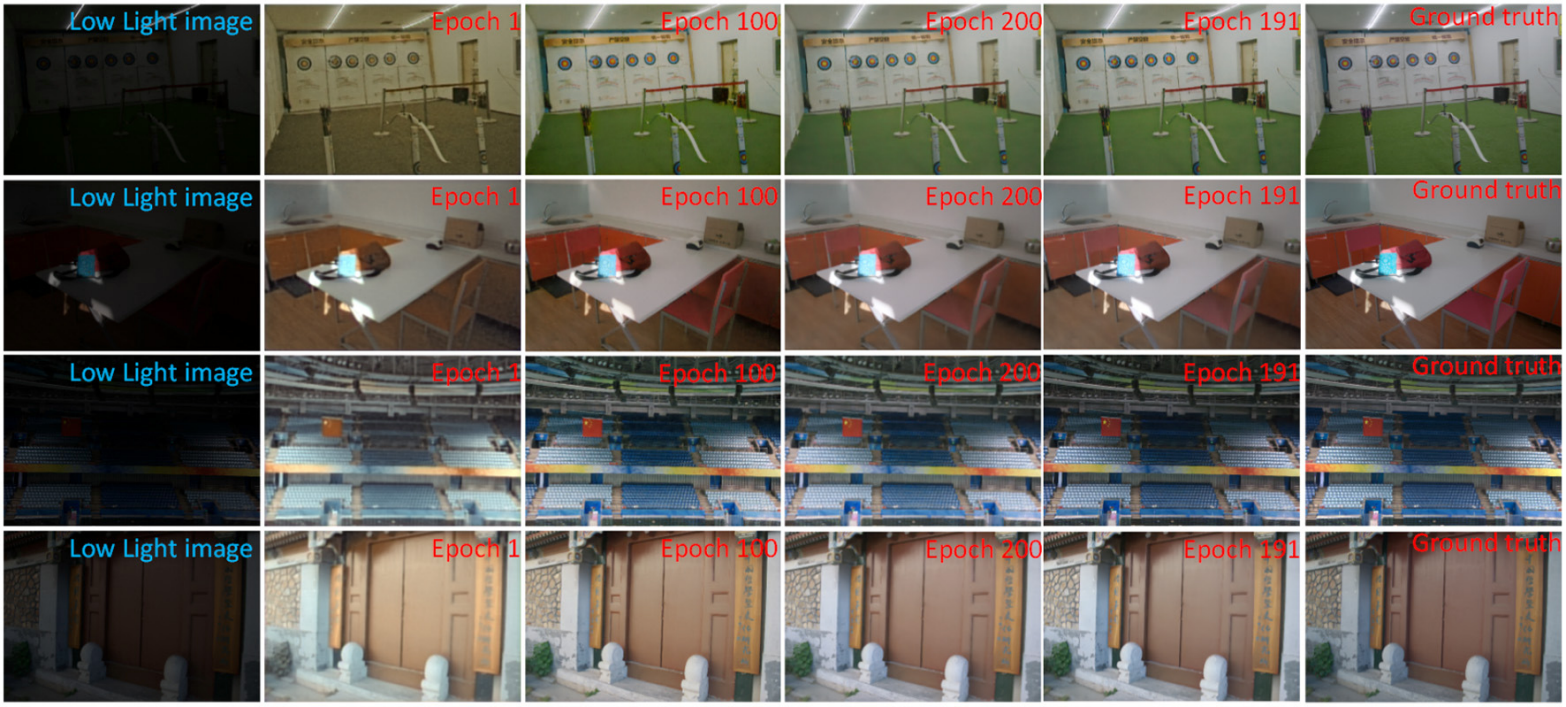
The enhancement results of different epoch validation sets during training.

In this article, the enhancement network is implemented based on PyTorch 0.4.0. The environment configuration used in the experiment is Ubuntu18.04 operating system with 64 GB DDR4 memory, with NVIDIA TITAN RTX graphics card, and CPU is E6-2640V4. The experimental procedure was optimized using ADAM with the initial learning rate set to 0.0001, and the learning rate decreased by 0.5 after 200 training periods. The whole network was trained for 300 periods, where the enhanced image was closest to the real image at epoch=191. Figure 6 shows the output images of different epoch validation sets, the training dataset consists of the LOL dataset (Wei et al., 2018) and the brightening train dataset in Wei et al. (2018). The LOL dataset has 500 images and the brightening train dataset has 1,000 images. The LOL dataset and the brightening train dataset are two datasets as open source datasets. Both datasets are datasets with true labels, i.e., each low light image has a normal light image corresponding to it. When the two data are combined, we do not perform any preprocessing and use the original dataset directly. The training set consists of 1,500 images. The training dataset consists of 1,300 training images, 100 validation images, and 100 test images. The image size of the training dataset is 256 and the batch size is 4. During the training process, we record the enhancement results for different epochs, as shown in Figure 6. At epoch=191, the enhanced images are more effective and the contrast and brightness of the images reach the best.

VV, MEF, DICM are name of three dataset. DICM dataset, This dataset contains 69 low-light images captured by commercial digital cameras. MEF dataset: This dataset is a multi-exposure image set. The dataset contains 17 high-quality image sequences with image styles including natural landscapes, indoor and outdoor landscapes, and man-made buildings. VV dataset: The dataset consists of 26 images collected from the daily life of Vassilios Vonikakis. In addition, we used the public datasets VV (Vonikakis et al., 2012), MEF (Ma et al., 2015), DICM (Lee et al., 2013), and LOL (Wei et al., 2018) for the testing of comparative experiments to demonstrate the effectiveness of the method proposed in this article. The VV dataset has 26 images, the MEF dataset has 17 images, the DICM dataset has 64 images, and the LOL dataset has 100 images.

The dataset consists of 26 images collected from the daily life of Vassilios Vonikakis (VV) (Vonikakis et al., 2012). This dataset is a multi-exposure image set. The dataset contains 17 high-quality image sequences with image styles including natural landscapes, indoor and outdoor landscapes, and man-made buildings (MEF) (Ma et al., 2015), This dataset contains 69 low-light images captured by commercial digital cameras (DICM) (Lee et al., 2013), and LOL (Wei et al., 2018) for the testing of comparative experiments to demonstrate the effectiveness of the method proposed in this article. The VV dataset has 26 images, the MEF dataset has 17 images, the DICM dataset has 64 images, and the LOL dataset has 100 images.

### 4.2. Subjective Evaluation

**Figure 7:**
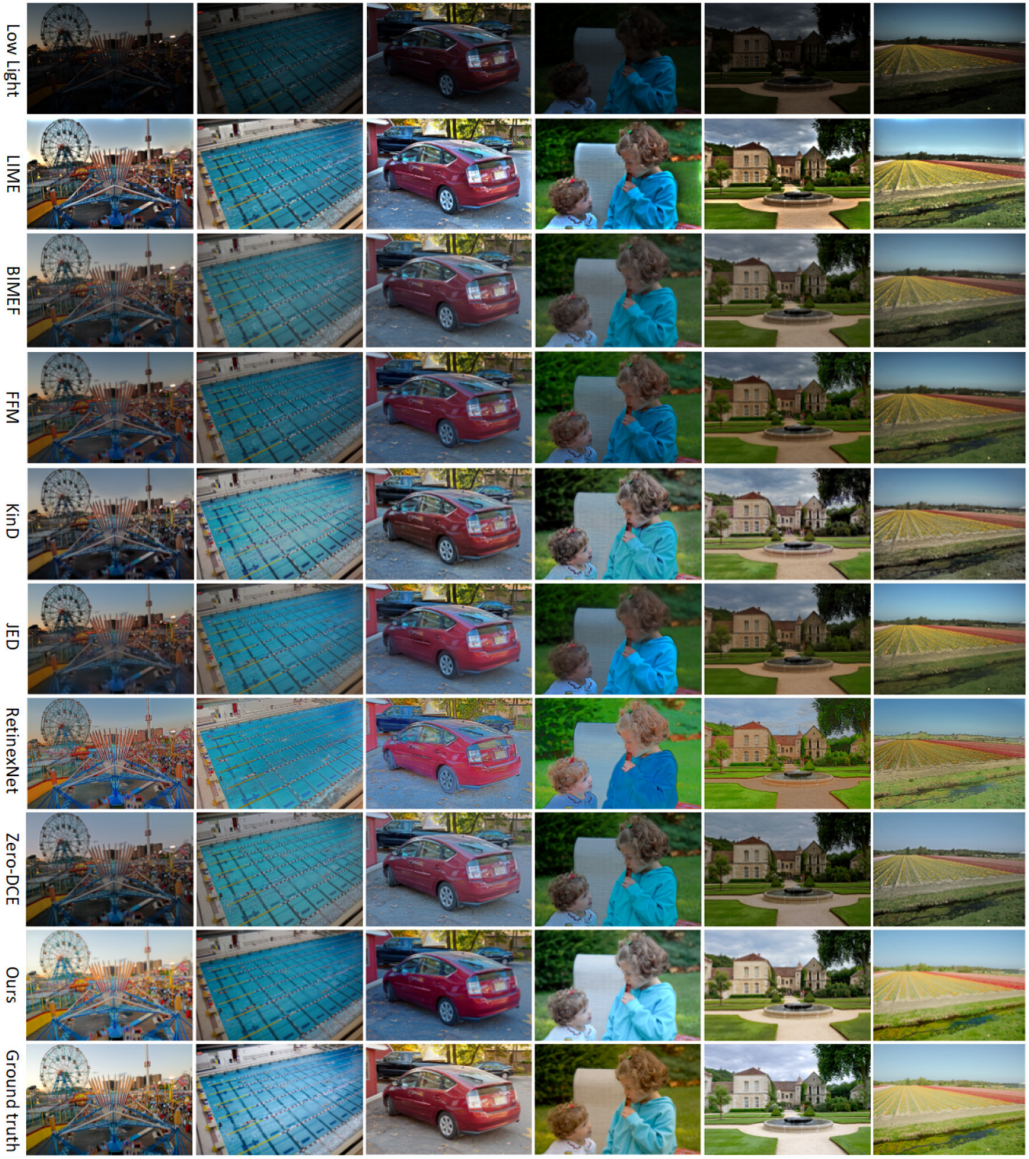
Rows 2 to 8 in the figure show the enhancement effect of seven existing methods on low-light images, and the enhancement effect of the method in this article in the ninth row.

We randomly selected six images from four public datasets, which include outdoor night, indoor, long-distance, near-distance, and people images, and we compared the enhancement results of different methods with seven existing commonly used shimmer image enhancement methods LIME (Guo et al., 2016), FFM (Dai et al., 2019), KinD (Zhang et al., 2019), JED (Ren et al., 2018), RetinexNet (Wei et al., 2018), BIMEF (Ying et al., 2017), and Zero-DCE (Guo et al., 2020), as shown in Figure 7.

In Figure 7, the first row is the input low-light image, and rows 2–9 are the enhanced images from LIME, BIMEF, FFM, KinD, JED, RetinexNet, Zero-DCE, and this article. In the second row, the result of the LIME method enhancement, the brightness is on the bright side, the image color is too deep, there are areas in the edge areas that are too bright, and the objects in the photo look vague and unrealistic. In the third row, the result of BIMEF method enhancement, the overall brightness of the image is dark, the image looks like a kind of foggy feeling, and some images have large noise, such as the gray shadow in the upper right corner of the second image pool. In the fourth row, the enhancement result of the FFM method, the sharpness and color of the image are relatively good, but the enhancement effect is not satisfactory for smaller objects, such as the flowers in the grass in the fifth picture, the brightness and color of the flowers are restored, but the shape of the flowers is enlarged. The fifth line, the result of KinD method enhancement, the overall brightness of the image is better, but some of the image color distortion, some areas appear some noise, such as the sixth picture, the color of the whole grass becomes gray, and the fourth picture of the character's face appears some black spots. In the sixth row, the result of the JED method enhancement, the image loses a lot of texture information and the details become blurred, such as the hair part of the person in the fourth image, in addition, the boundary between the person and the background in the image is blurred. In the seventh row, the enhancement result of RetinexNet method, the image brightness is over-enhanced, the colors are distorted and unevenly distributed, the edges of the object are defocused, and the texture details of the object are severely lost. In the eighth row, the enhancement results of the Zero-DCE method, which has relatively better enhanced images compared to the previous methods, but the images still have some artifacts, such as the edge of the roof in the fifth image and the outline of the car in the third image. In the ninth row, the enhancement results of the proposed method in this article, the images have normal brightness, uniform color, clear objects, and rich texture detail information without artifacts.

**Figure 8:**
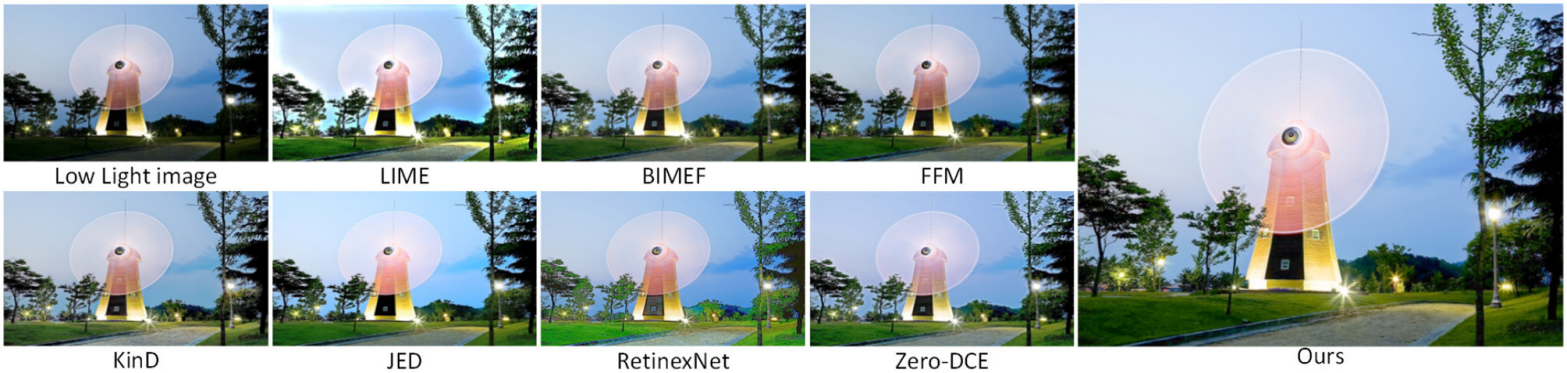
Comparison of various low light image enhancement methods using night images from the DICM dataset.

Figure 8 shows a scene of a building at night with image enhancement using the seven methods described above and our method. In low light images, the brightness of the sky and the trees around the buildings are dark and we do not see the details of the whole image clearly. The JED method in Figure 8 clearly improves the brightness of the sky, but the color recovery is not uniform. The RetinexNet method improves the brightness of the sky and trees, but the color of the clouds in the sky is distorted and the color of the surrounding lawn is over-enhanced. Compared with the previous methods, Zero-DCE enhancement is better, but there is less noise in the sky in the image. Compared with other methods, our method achieves a better enhancement effect.

**Figure 9:**
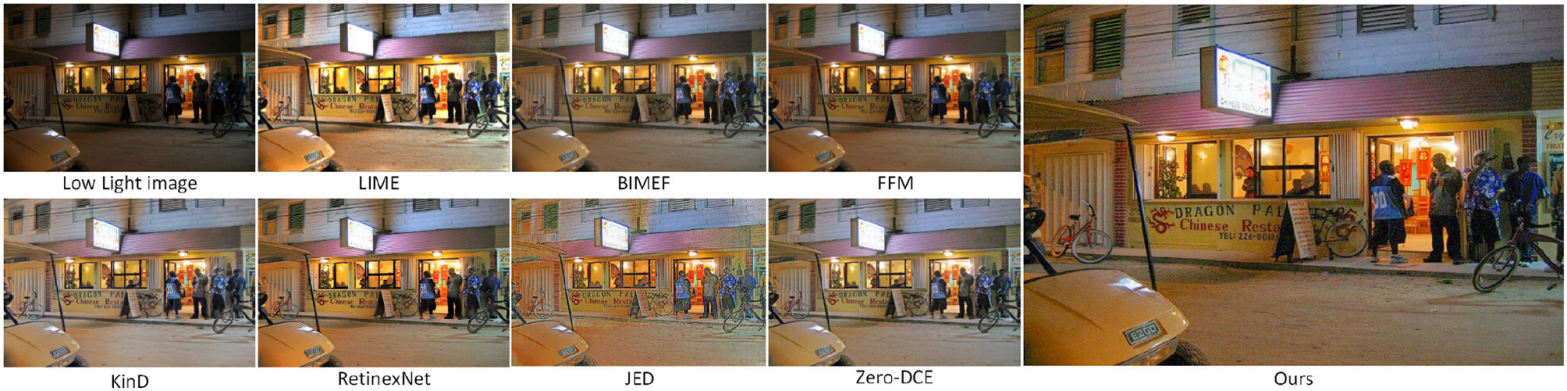
Comparison of various low light image enhancement methods using night images from the VV dataset.

In Figure 9, we enhance the night scene images in the VV dataset. In the original input image, the brightness of the area illuminated by the light is relatively high, while the brightness of the area farther away from the light is relatively low, and the phenomenon of over-enhancement of the brightness area can easily occur during the enhancement process. Compare 7 enhancement methods with our method, KinD enhancement results, the overall brightness of the image is increased, but the tone tends to be white, which does not meet the visual requirements when viewing. The enhancement result of the JED method is even less satisfactory, the whole image quality is blurred, the edges of the object are distorted, and the whole image has more noise. It can be clearly seen that the overall enhancement effect of our proposed method is better, with uniform image color, bright and clear objects, and not much noise.

**Figure 10:**
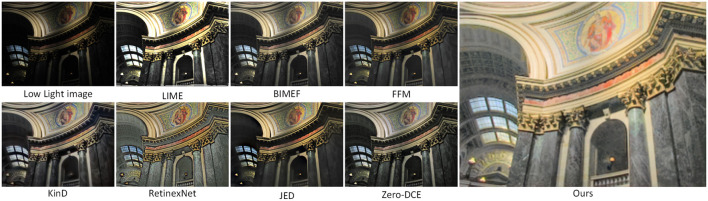
Comparison of various low light image enhancement methods using indoor images from the MEF dataset.

Figure 10 shows the enhanced results for the indoor scenes in the MEF dataset. In the original image, the brightness of all areas is dark except for the top of the attic, which is bright. The LIME method mainly enhances the brightness area in the original image, and you can see that only the top of the loft is over-enhanced. The KinD method enhances the image with rich detail information and clear object texture, but the brightness of the image needs to be improved. RetinexNet and JED enhancement results are blurred, the enhancement results of this article's method, the entire top of the attic have uniform color, normal brightness, and the windows and carved patterns on the top of the attic are particularly clear. On the whole, the effect of this article's method is significantly better than other methods.

### 4.3. Quantitative Evaluation

In order to analyze the enhancement effects of various methods more objectively and rigorously, we used image quality evaluation metrics in addition to the subjective evaluation above. We use four image quality evaluation metrics: peak signal-to-noise ratio (PSNR), structural similarity (SSIM) (Loh and Chan, 2018), the blind/referenceless image spatial quality evaluator (BRISQUE) (Mittal et al., 2012) and the natural image quality evaluator (NIQE) (Mittal et al., 2013).

PSNR evaluates the image quality by calculating the error between pixel points. When the error between the image enhancement and the original image is less, the larger the PSNR value is, the better the image enhancement is.

SSIM is used to measure the similarity between the original low-light image and the reconstructed clear image. SSIM uses the mean for luminance estimation, SD for contrast estimation, and covariance for a measure of structural similarity. The larger the SSIM value, the less distorted the image, and the better the reconstruction.

BRISQUE is a reference-free image evaluation method that scores by comparing the differences between the test image and the natural image. BRISQUE uses locally normalized luminance coefficients to obtain the corresponding parametric features, and compares the features of the test image with the standard natural image. When the difference is large, the higher the BRISQUE score is, the worse the image quality is.

NIQE is also a reference-free image evaluation method. NIQE extracts spatial domain features on the test image and describes the spatial domain features using a Gaussian distribution. The features of the test image are compared with the standard natural image. The larger the NIQE value, the larger the difference between the test image and the standard natural image, and the poorer the image enhancement effect.

For the six images in Figure 7, the average performance of each low-light image enhancement method, the results are shown in Table 1. In order to show the effectiveness of the model in this article, we choose the public dataset LOL (Wei et al., 2018) with real labels and use the evaluation metrics: four metrics PSNR, SSIM (Loh and Chan, 2018), NIQE (Lee et al., 2013), and BRISQUE (Ma et al., 2015) on this dataset for quantitative evaluation, and the results are shown in Table 2, and the values are the average of each metric. From Table 2, it can be seen that our method outperforms the other methods for all four metrics. For the non-reference datasets VV (Vonikakis et al., 2012), MEF (Ma et al., 2015), and DICM (Lee et al., 2013), we use the non-reference indicators BRISQUE and NIQE for indicator estimation. Table 3 shows the average performance of the various methods on the three data sets. The BRISQUE scores of the BIMEF method are better in the MEF dataset, and the metrics of the LIME method are better than several other methods in the DICM dataset and the VV dataset, but our method shows better BRISQUE scores in the MEF, DICM, and VV datasets. For the NIQE metric, the BIMEF method works better in the MEF dataset, and the NIQE score is better than the proposed method in this article, but in the DICM and VV datasets, our method shows better performance. It can be seen from the three tables that our method has better average performance in most of the datasets.

**Table 1:** The average performance of the various enhancement methods in Figure 7, with the best results labeled in red and the second-best results in color.

**Method**	**PSNR**	**SSIM**	**NIQE**	**BRISQUE**
LIME (Guo et al., 2016)	17.0178	0.0.8252	4.8983	18.6163
BIMEF (Ying et al., 2017)	13.9948	0.8156	4.4113	18.4749
FFM (Ren et al., 2018)	12.0758	0.7443	4.4725	18.4066
KinD (Dai et al., 2019)	18.1828	0.8457	4.6839	18.5455
JED (Zhang et al., 2019)	14.6821	0.7603	5.5213	18.7889
RetinexNet (Zhang et al., 2019)	15.7293	0.7920	5.5280	18.8763
Zero-DCE (Guo et al., 2020)	16.3743	0.8709	4.6208	18.5193
Ours	21.6257	0.9191	4.1998	18.4016

**Table 2:** Quantitative evaluation of the low light paired dataset (LOL) dataset, with the best results marked in red and the second best results marked in blue.

**Method**	**PSNR**	**SSIM**	**NIQE**	**BRISQUE**
LIME (Guo et al., 2016)	18.4083	0.5840	8.4091	46.3440
BIMEF (Ying et al., 2017)	18.5550	0.7233	7.7284	30.0861
FFM (Ren et al., 2018)	17.6991	0.7680	6.9621	28.7708
KinD (Dai et al., 2019)	16.5800	0.5765	4.7219	28.9662
JED (Zhang et al., 2019)	18.0364	0.7725	5.7988	29.4081
RetinexNet (Zhang et al., 2019)	14.5686	0.2990	9.4277	33.8642
Zero-DCE (Guo et al., 2020)	18.9855	0.6768	8.0578	30.1077
Ours	22.1014	0.8886	4.6278	20.9614

**Table 3:** BRISQUE and NIQE quantitative evaluations of the public datasets MEF, DICM, VV, and LIME, with the best results labeled in red font and the second best results labeled in blue.

	**BRISQUE**			**NIQE**		
**Method**	**MEF**	**DICM**	**VV**	**MEF**	**DICM**	**VV**
LIME Guo et al. (2016)	23.3407	18.4335	20.2027	5.7189	3.3993	3.8025
BIMEF (Ying et al., 2017)	20.5770	24.2961	23.1839	4.3061	3.8263	3.6850
FFM Ren et al. (2018)	25.5495	25.6837	29.4840	4.7857	3.7660	3.5581
KinD Dai et al. (2019)	25.9532	24.9554	24.5015	5.6242	4.1372	3.5892
JED Zhang et al. (2019)	25.1958	28.3969	29.3638	6.4179	3.9760	4.8492
RetinexNet Zhang et al. (2019)	24.9352	27.5574	25.8767	6.1001	4.4492	4.1033
Zero-DCE Guo et al. (2020)	24.5455	24.8596	22.7712	4.4834	4.5601	3.5707
Ours	21.7495	18.0490	18.8505	4.4113	3.7634	3.3672

*On the MEF dataset, our low-light image enhancement method is slightly inferior to the BIMEF low-light image enhancement when we perform comparison experiments using the NIQE metric. Where RN is RetinexNet and ZD is Zero-DCE*.

### 4.4. Ablation Experiments

**Figure 11:**
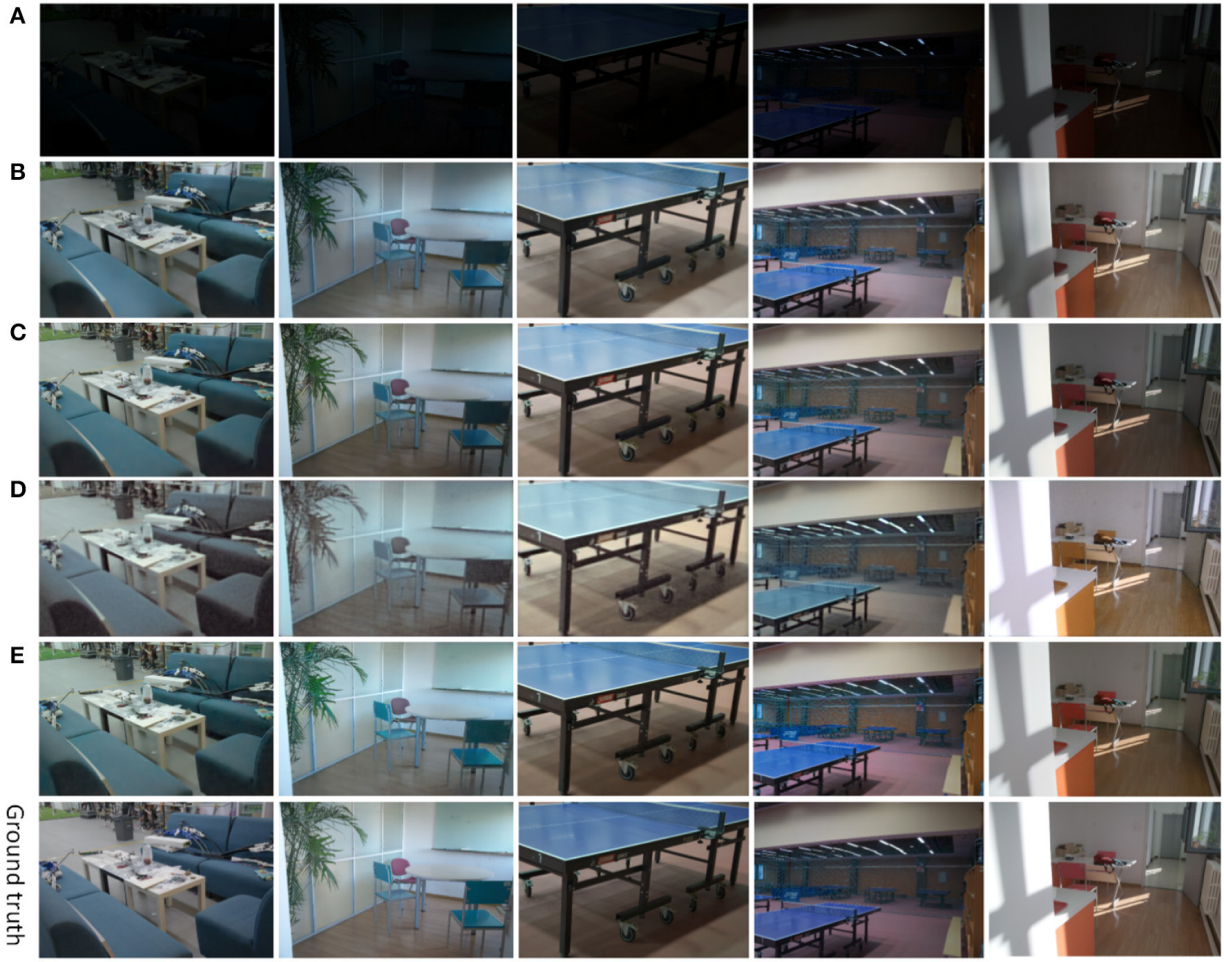
Results of ablation experiments **(A)** original images of the input, **(B)** enhanced results of experiment 1, **(C)** enhanced results of experiment 2, **(D)** enhanced results of experiment 3, and **(E)** our method.

**Figure 12:**
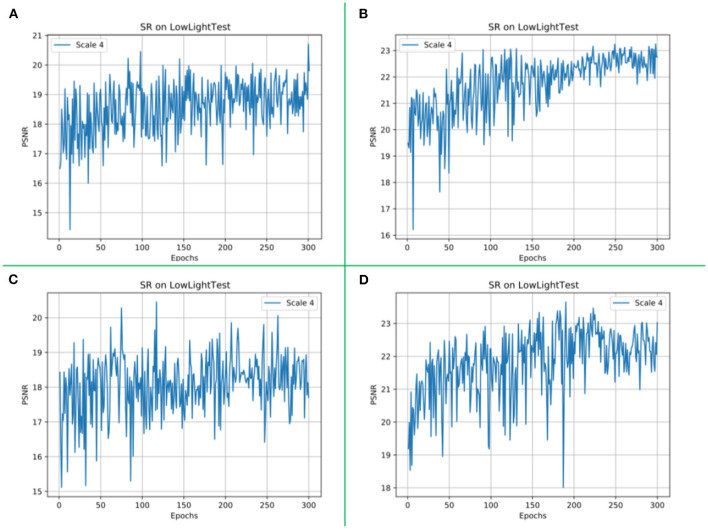
The PSNR values of the validation dataset at different epochs during training. Experiment 1 is shown as **(A)** in this figure. Experiment 2 is shown as **(B)** in this figure. Experiment 3 is shown in **(C)** in this figure. The PSNR value of our proposed method is shown in **(D)** in this figure.

The network model proposed in this article adds the CBAM module and inception module. In order to verify the necessity and rationality of these two modules, we conduct two groups of ablation experiments. In the ablation experiment, the control variable method is used, and the variable control is shown in Table 4, and the corresponding image visual effects of each group of experiments are shown in Figure 11. The values of the image quality evaluation index PSNR corresponding to each group of experiments are shown in Figure 12.

**Table 4:** Control of variables in the ablation experiment, where 0 means that the corresponding module is not used and 1 means that the corresponding module is used.

**Variable**	**Experiment1**	**Experiment2**	**Experiment3**	**Experiment4**
CBAM	1	0	0	1
Inception	0	1	0	1

In addition, we recorded and displayed the PSNR values of the validation sets of experiments 1, 2, 3, and 4 on different epochs, as shown in Figure 12.

In Experiment 1, shown in Figure 11B. We did not use the CBAM module. The input is an image with dark luminance, and the initial learning rate is set to 0.0001, and the learning rate decreases by 0.5 after 200 periods of training, and the whole network is trained for 300 periods, and the enhancement results are shown in Figure 11B. It can be seen that in the first image in (b), the sofa has a dark color and a rough surface. The floor of the room is dark in color, and the overall brightness of the image is low, with low contrast and saturation. In the third image, under normal circumstances, in the table tennis, table top color should be blue, but the recovered color is blue-gray.

In Experiment 2, as shown in Figure 11C, we did not use the inception module. The input is an image with dark luminance, the initial learning rate is set to 0.0001, the learning rate decreases by 0.5 after 200 periods of training, and the whole network is trained for 300 periods. The enhancement results are shown in Figure 11C. Removing the inception module has little effect on the visual effect of the image. It can be seen that the surface of the sofa in (c) is smoother than that in (b), the ground color is brighter, and the color of the enhanced image is slightly distorted.

In experiment 3, as shown in Figure 11D, we neither did use the CBAM module, nor did we use the inception module. The initial learning rate was set to 0.0001, and the learning rate decreased by 0.5 after 200 training periods, and the enhancement results for the whole network trained for 300 periods are shown in Figure 11D. Compared with the first two ablation experiments, the enhancement results of experiment 3 have changed significantly. We can clearly see that the color of the sofa in the first image is completely distorted and a large amount of noise appears on the surface. The enhancement effect of other objects around the sofa is even more desirable. In the second image, the clarity is lower, the texture details are recovered incompletely, and the image as a whole presents a blurred visual effect. In the third image, the color of the table is mixed with the color of the wall, and the presence of the table is completely invisible without careful observation.

In Experiment 4, as shown in Figure 11E, the method proposed in this article is used, using both the CBAM module and the inception module. The initial learning rate is set to 0.0001, and the learning rate decreases by 0.5 after 200 training periods, and the whole network is trained for 300 periods to enhance the results as shown in Figure 11E. Compared with the first three experiments, the surface of the couch is smooth, free of noise, and with clear edges. In the second image, the green leaves and chairs are brightly colored, and there is no problem with excessive color intensity. The table top of the ping pong table in the third and fourth images is a normal blue color. It can be seen that the method proposed in this article enhances the image with more realistic and natural colors more uniformly, with normal brightness, clear objects, and good visual effects.

## 5. Conclusion

In this article, we propose a recursive structure-based image enhancement network. Existing image enhancement methods are prone to color distortion or poor contrast, which seriously affects other computer vision tasks. In order to acquire high-quality images, we combine the dual attention module CBAM, U-Net module, and design a recurrent recursive network structure. The recursive network can use the previously enhanced information as a priori information and use the priori information to correct the deficiencies in the learning process of the network during the current recursion. In the network, the CBAM module is used to extract channel and spatial feature attention maps, which circumvents the drawback of focusing only on important features at the channel level or only on important features in the spatial dimension. Without significantly increasing the number of network parameters, we use the inception model to expand the width and depth of the U-Net network to form the MIU module. The MIU module extracts and fuses multi-scale features, and this module reduces the number of network parameters without reducing the enhancement capability of the network. In the experimental section analysis, the subjective and objective analysis of our method is performed. The results show that our method achieves better performance in several evaluation metrics. Finally, we perform ablation experiments to demonstrate the effectiveness of each modular structure in the network. In our future research plan, we aim to focus on improving the U-Net model to achieve a better low-light image enhancement algorithm.

## Data Availability Statement

The raw data supporting the conclusions of this article will be made available by the authors, without undue reservation.

## Author Contributions

All authors listed have made a substantial, direct, and intellectual contribution to the work and approved it for publication.

## Funding

This research was supported by the National Natural Science Foundation of China (61772319, 62002200, 12001327, 62176140), Shandong Natural Science Foundation of China (ZR2020QF012 and ZR2021MF068).

## Conflict of Interest

The authors declare that the research was conducted in the absence of any commercial or financial relationships that could be construed as a potential conflict of interest.

## Publisher's Note

All claims expressed in this article are solely those of the authors and do not necessarily represent those of their affiliated organizations, or those of the publisher, the editors and the reviewers. Any product that may be evaluated in this article, or claim that may be made by its manufacturer, is not guaranteed or endorsed by the publisher.
